# Mechanistic studies of a novel C-S lyase in ergothioneine biosynthesis: the involvement of a sulfenic acid intermediate

**DOI:** 10.1038/srep11870

**Published:** 2015-07-07

**Authors:** Heng Song, Wen Hu, Nathchar Naowarojna, Ampon Sae Her, Shu Wang, Rushil Desai, Li Qin, Xiaoping Chen, Pinghua Liu

**Affiliations:** 1 Departments of Chemistry, Boston University, Boston, MA 02215, USA; 2 State Key Laboratory of Respiratory Diseases, Center for Infection and Immunity, Guangzhou Institutes of Biomedicine and Health, Chinese Academy of Sciences, Guangzhou, 510530, China

## Abstract

Ergothioneine is a histidine thio-derivative isolated in 1909. In ergothioneine biosynthesis, the combination of a mononuclear non-heme iron enzyme catalyzed oxidative C-S bond formation reaction and a PLP-mediated C-S lyase (EgtE) reaction results in a net sulfur transfer from cysteine to histidine side-chain. This demonstrates a new sulfur transfer strategy in the biosynthesis of sulfur-containing natural products. Due to difficulties associated with the overexpression of *Mycobacterium smegmatis* EgtE protein, the proposed EgtE functionality remained to be verified biochemically. In this study, we have successfully overexpressed and purified *M. smegmatis* EgtE enzyme and evaluated its activities under different *in vitro* conditions: C-S lyase reaction using either thioether or sulfoxide as a substrate in the presence or absence of reductants. Results from our biochemical characterizations support the assignment of sulfoxide **4** as the native EgtE substrate and the involvement of a sulfenic acid intermediate in the ergothioneine C-S lyase reaction.

Glutathione, one of the most abundant natural thiols inside the cells (up to 10 mM), plays a key role in buffering the intracellular redox-state. In many organisms, there exists another important thiol, ergothioneine, which is a thio-imidazole containing amino acid (**5**, Fig. 1)^1^,^2^,^3^ Different from glutathione, the predominant form of ergothioneine is its thione form (**5b**, Fig. 1). As a result, ergothioneine’s reduction potential (E^0^ = −0.06 V)^2^ is significantly higher than that of glutathione (E^0^ = −0.24 V)^4^,^5^ Humans do not synthesize ergothioneine. However, through an ergothioneine-specific transporter (OCTN1), we enrich ergothioneine from our diets to mM concentrations in many parts of our body^6^, including liver, kidneys, central nervous system, erythrocytes, eye lenses, and seminal fluids^2^,^7^,^8^,^9^,^10^. Ergothioneine has many beneficial roles to human health^2^,^4^,^11^,^12^, especially its role as an effective scavenger for reactive oxidative species (ROS), including singlet oxygen, hydroxyl, peroxyl, peroxynitrite (ONOO^-^), nitrosoperoxycarbonate (ONOOCO_2_^−^), and carbonate radicals^13^,^14^,^15^,^16^.

Due to ergothioneine’s beneficial roles to human health, biochemists have been searching for the ergothioneine biosynthetic pathway since the 1960 s^17^,^18^,^19^. The ergothioneine biosynthetic genes were discovered only very recently and there exists two different ergothioneine biosynthetic pathways (Fig. 1)^20^,^21^,^22^,^23^. In 2010, the ergothioneine biosynthetic gene cluster in *Mycobacterium smegmatis* was discovered^20^. The mycobacterial pathway involves five steps: EgtD catalyzes the methylation of histidine to hercynine (**2**); EgtA condenses glutamate and cysteine to form γ-glutamylcysteine (γ-Glu-Cys, **6**); EgtB is a non-heme iron enzyme, catalyzing oxidative coupling of hercynine (**2**) and γ-Glu-Cys (**6**) to introduce the thio-functionality to the histidine side-chain; EgtC is an amidotransamidase, hydrolyzing glutamate from **3** to produce **4**; EgtE is proposed to be a PLP-dependent C-S lyase. In contrast, in the fungal ergothioneine biosynthetic pathway, Egt1 enzyme catalyzes a one-step **2** → **4** transformation^20^,^21^,^22^,^23^. As a result, ergothioneine biosynthesis in fungi does not compete with glutathione biosynthesis (Fig. 1).

When the *M. smegmatis* ergothioneine biosynthetic gene cluster was discovered, the *in vitro* activities of EgtA, EgtB, EgtC, and EgtD enzymes were verified. However, the proposed C-S lyase activity (EgtE) was not demonstrated *in vitro* due to difficulties in *M. smegmatis* EgtE overexpression. In this study, we report the isolation of EgtE protein and the detailed biochemical characterization of its novel C-S lyase activity. EgtE makes use of both sulfoxide (**4**) and thio-ether (**8**, Fig. 2) as substrates. In addition, different outcomes were observed when sulfoxide (**4**) was used as the substrate in the absence or in the presence of reductants. More in-depth kinetic characterizations suggest that sulfoxide **4** is the biological EgtE substrate. More importantly, subsequent studies led to the trap of a sulfenic acid intermediate in EgtE-catalysis. Small molecular sulfenic species are highly reactive in nature and stable small molecular sulfenic species are rare. The unique ergothioneine chemical property may stabilize the sulfenic species and explains our successful trapping of this species in EgtE reaction.

## Results

We sub-cloned *M. smegmatis* EgtE gene into the pASK-IBA3^+ ^vector and the protein was overexpressed in the *E. coli* BL21(DE3) strain. The expressed EgtE was then purified using Strep-Tactin resin to near homogeneity (higher than 95% based on the analysis of the SDS-PAGE, supplementary Fig. 1). Furthermore, as suggested by EgtE sequence analysis, its UV-visible spectra are consistent with the presence of a PLP cofactor (Fig. 3). The pH-dependence of the EgtE internal aldimine electronic absorption was also studied. In PLP-containing proteins, the PLP-Lysine conjugate can exist in a few different forms: enolimine (**9**), ketoenamine (**10**) or deprotonated ketoenamine (**11**)^24^,^25^,^26^,^27^,^28^,^29^,^30^,^31^. For the isolated EgtE protein at pH 7.5, it has an absorption feature near 420 nm (Supplementary Fig. 1), which is consistent with the presence of ketoenamine (**10**) tautomer of the protonated Schiff base. As the pH changes from 6.0 to 10.0, the UV-vis spectra (Fig. 3) do not show distinct changes in the overall absorption features and the maximal absorption wavelengths. Thus, for EgtE, the internal aldimine is present mainly in the form of **10**. The amount of PLP per EgtE monomer was also determined after it was released by 0.2 M NaOH treatment^32^. The isolated EgtE has 0.8 mole PLP per mole of EgtE monomer.

Several PLP-dependent C-S lyases (e.g., cysteine *S*-conjugate β-lyases) in primary metabolisms, xenobiotics detoxifications^33^, and secondary metabolite biosynthesis have been reported^34^,^35^. There are two types of C-S lyases in the literatures^1^,^2^,^3^. Most literature examples use thioethers as substrates. A few sulfoxide utilizing C-S lyases were also noted. In literature, C-S lyase activities in ergothioneine biosynthesis have been examined using *M. smegmatis* cell lysate^36^. When cell lysate was used as the catalytic system, in the absence of reductants, if sulfoxide (**4**) is the substrate, ergothioneine was not produced. In the absence of reductants, thioether does lead to ergothioneine production. In addition, C-S lyase activity was observed even with PLP alone. All of these C-S lyase activities are at very low levels. Thus, it remained to be verified whether sulfoxide **4** or thio-ether **8** (Fig. 2) is the EgtE native substrate and how much EgtE can accelerate the reaction rate relative to the catalysis by PLP alone. To address these issues, we synthesized both compounds **4** and **8** (Fig. 2). Compound **4** was synthesized using our newly discovered enzymatic synthetic method^37^. The OvoA enzyme from the ovothiol biosynthetic pathway^38^ or the Egt1 enzyme from the fungal ergothioneine biosynthetic pathway^21^ can catalyze a direct oxidative coupling between hercynine and cysteine to form sulfoxide **4** (Fig. 2)^21^,^23^,^37^. Thio-ether **8** was synthesized by following a recently reported chemical synthetic method (Fig. 2, Supplementary Fig. 2–4)^39^.

**Figure 1:**
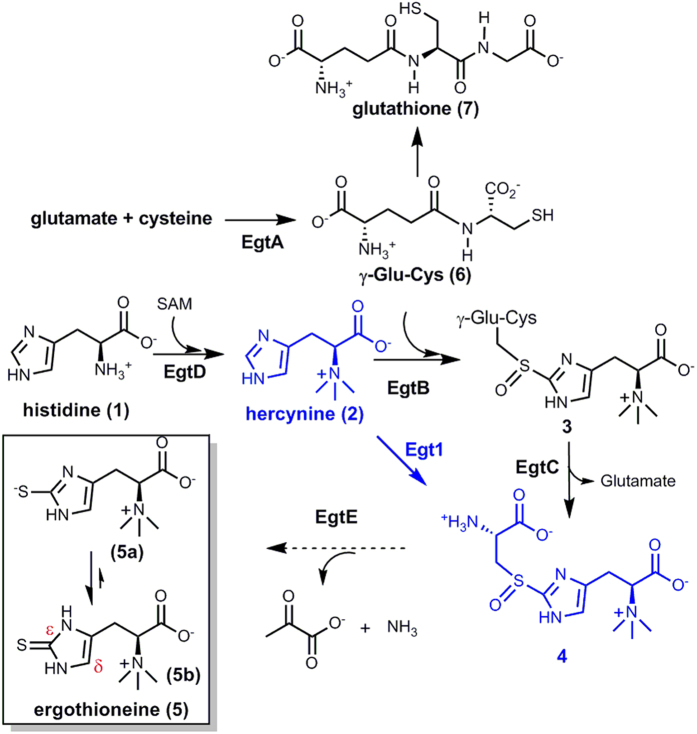
Two different ergothioneine biosynthetic pathways. (**A**) The *M. Smegmatis* ergothioneine biosyntetic pathway (EgtA-EgtE catalysis). (**B**) The fungal *N.crassa* ergothioneine biosynthetic pathway (Egt1).

**Figure 2:**
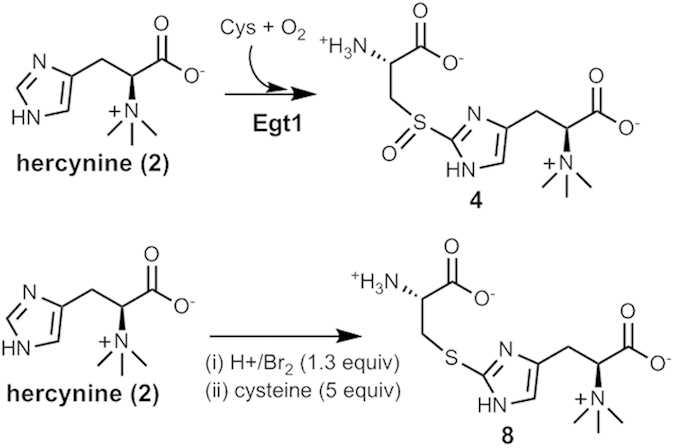
Enzymatic and chemical syntheses of EgtE substrate candidates (4 & 8).

**Figure 3:**
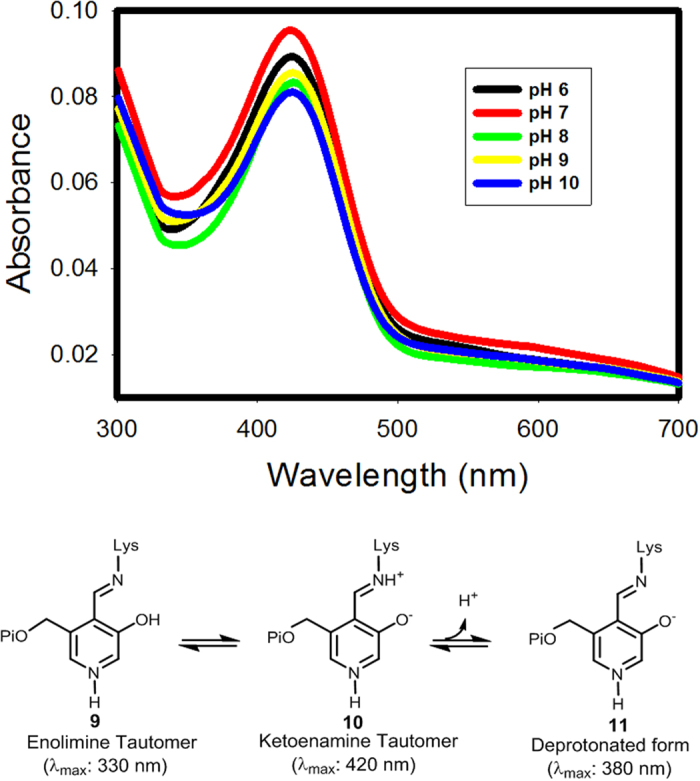
UV-visible spectra of EgtE (30 μM) at a few different pHs.

**Figure 4:**
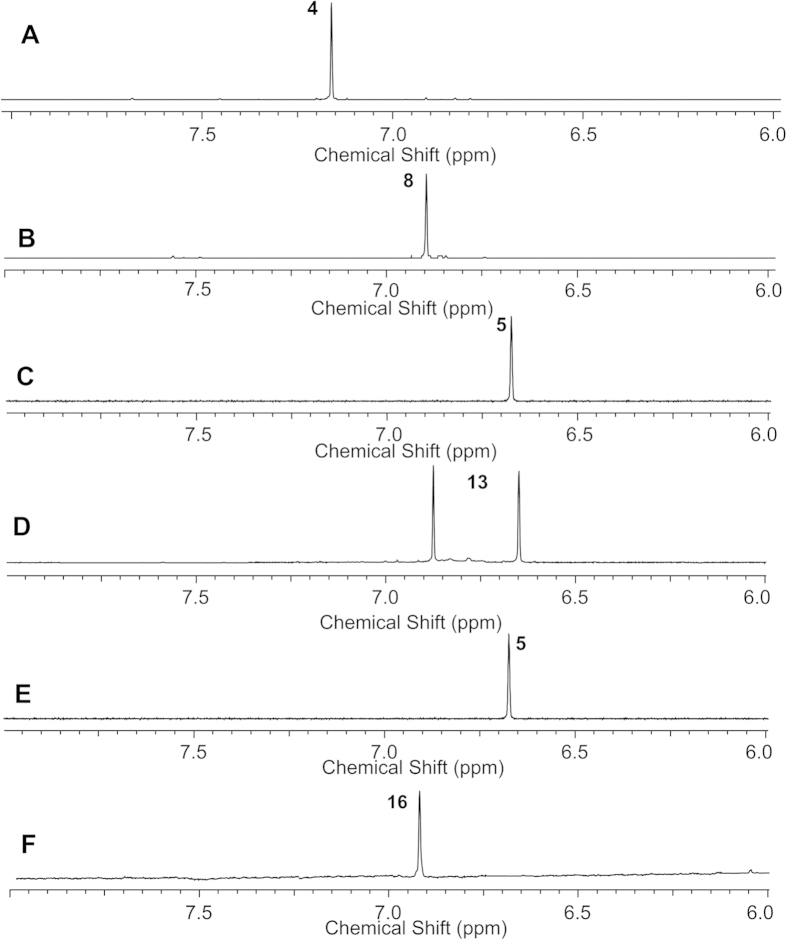
^1^H-NMR spectrum of EgtE reactions using either thio-ether 8 or sulfoxide 4 as the substrate. (**A**) Pure sulfoxide **4**; (**B**) Pure thio-ether **8**; (**C**) EgtE reaction using thio-ether **8** as the substrate; (**D**) EgtE reaction using sulfoxide **4** as the substrate; (**E**) EgtE reaction using sulfoxide **4** as the substrate and in the presence of DTT as the reductant; (**F**) EgtE reaction using sulfoxide **4** as the substrate and in the presence of 50 × of 1,3-cyclohexanedione.

**Figure 5:**
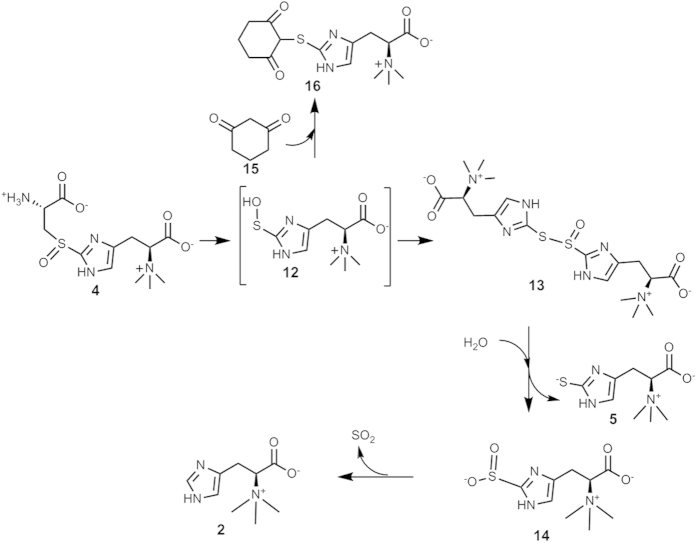
Proposed model to explain EgtE reaction outcomes when sulfoxide 4 was the substrate.

After the EgtE substrate candidates (**4** & **8**) were synthesized, ^1^H-NMR was used to analyze EgtE reaction directly by monitoring the chemical shift of the hydrogen atoms on the hercynine imidazole side-chains in the substrate (**4** or **8**) and in the product, ergothioneine (**5**, Fig. 4). In ^1^H-NMR spectrum, the imidazole hydrogen in sulfoxide **4** has a chemical shift of 7.15 ppm, while the imidazole hydrogen in thio-ether **8** has a chemical shift of 6.89 ppm. Once the C-S bond is cleaved by EgtE, the resulting ergothioneine thiol-imidazole has a chemical shift of 6.67 ppm. Surprisingly, different from the assignment of EgtE function (Fig. 1)^20^, our ^1^H-NMR assay clearly indicates that EgtE recognizes both sulfoxide **4** and thio-ether **8** as substrates. This is consistent with the studies using *M. smegmatis* cell lysate reported by Khonde *et al.*^36^ However, many fine details were revealed when pure EgtE was used. When thio-ether **8** was used as the substrate, EgtE converts it to ergothioneine very efficiently (Fig. 4C). When sulfoxide **4** was used as substrate in KPi buffer, EgtE indeed accepts compound **4** as a substrate. However, ^1^H-NMR assay does not support the production of ergothioneine as the product (Fig. 4D). Instead, there are two signals at the imidazole ring hydrogen chemical shift region. Closer examination of the ^1^H-NMR spectrum of EgtE reaction mixture (Supplementary Fig. 5) revealed that there are also two sets of α-protons and imidazole ring protons. 2D-gCOSY NMR spectroscopy was then adopted to further characterize these two products. Both H-5 and H-5′ show correlation to the β-proton (H-3 and H-3′), which suggests two different kinds of histidine derivatives. Moreover, both sets of the α-protons (H-2 and H-2′) are correlated with the β-protons (H-3 and H-3′), which suggests that the histidine skeleton remains intact. When the products were isolated by HPLC, two products were identified, ergothioneine (**5**) and ergothioneine-2-sulfinic acid (**14**, Fig. 5). Both compounds were fully characterized by ^1^H-NMR, ^13^C-NMR, 2-D NMR, and high-resolution mass spectrometry (Supplementary Fig. 6-15).

During the isolation process, it was also discovered that ergothioneine-2-sulfinic acid (**14**) degraded to hercynine under acidic condition, which is consistent with the literature information. Ergothioneine-2-sulfinic acid (**14**) is not stable and will convert to hercynine through elimination of sulfur dioxide under acidic conditions^40^. When the EgtE reactions using either thio-ether **8** or sulfoxide **4** as substrate were compared, the difference in sulfur oxidation state suggests that redox-chemistries must be considered in order to explain the formation of ergothioneine and ergothioneine-2-sulfinic acid (**14**). To explain this result, we proposed that a sulfenic acid intermediate (**12**, Fig. 5) is involved when sulfoxide **4** is used as substrate. It is known that sulfenic acids are not stable and in the absence of other oxidants or reductants^3^,^41^, and they disproportionate to form the thiol ester of thio-sulfinic acid (**13**), which results in two sets of histidine derivative ^1^H-NMR signals (Fig. 4D and Supplementary Fig. 5). During the HPLC isolation process, thiol ester of thio-sulfinic acid **13** can be hydrolyzed to produce ergothioneine (**5**) and ergothineine-2-sulfinic acid (**14**). Recently, the behaviors of ergothioneine under oxidative conditions were examined^42^. The involvement of a sulfenic acid intermediate was suggested in order to explain the outcomes of ergothioneine oxidation reactions. Indeed, several products observed in EgtE studies reported here were also observed in ergothioneine oxidation reaction. The mechanistic model (Fig. 5) outlined here explains both the EgtE reaction results and the ergothioneine oxidative reactions, in which the most important feature is the involvement of a sulfenic acid intermediate (**12**).

In our subsequent experiments, two lines of evidence were provided to support the involvement of a sulfenic acid intermediate in EgtE catalysis (Fig. 5). First, in the above EgtE reaction using sulfoxide **4** as substrate, reductant was not included in the reaction mixture. If sulfenic acid (**12**) is indeed produced as the product, a reductant might be able to reduce it to ergothioneine directly, instead of going through the disproportionation process. To test this hypothesis, we repeated the EgtE reaction using compound **4** as substrate and included DTT as reductant. Indeed, in the presence of DTT, the only detectable product is ergothioneine (Fig. 4E). *In vivo*, natural thiols (e.g., cysteine, glutathione, or mycothiol) might serve as reductant to fulfill this role.

To provide further evidence supporting the presence of sulfenic acid intermediate **12** (Fig. 5) in EgtE catalysis, we have attempted trapping this intermediate. Protein cysteine residue-based sulfenic acid has been suggested to be a key intermediate in oxidative stress-related signaling^43^,^44^,^45^,^46^. The detection of thiol ester of thio-sulfinic acid **13** in EgtE reaction suggests that ergothioneine sulfenic acid intermediate (**12**) might be released from the enzyme active site because the formation of thio-sulfinic acid (**13**) involves two molecules of ergothioneine sulfenic acid (**12**). Among the reported sulfenic acid trapping methods^47^,^48^,^49^, the dimedone method was examined in order to trap the proposed ergothioneine sulfenic acid intermediate. The EgtE reaction was conducted using sulfoxide **4** as substrate in the absence of reductants and by including 50 × of 1,3-cyclohexanedione as the trapping reagent relative to the sulfoxide **4** concentration. Different from the reaction without dimedone (Fig. 4D), which has two signals (6.65 ppm and 6.87 ppm) in the imidazole hydrogen chemical shift region, when 50 × of 1,3-cyclohexanedione was included in the reaction mixture, only one signal (6.93 ppm) was observed (**16**, Fig. 4F). The different EgtE reaction outcomes between these two reaction conditions (Fig. 4D vs. 4F) highly suggest that 1,3-cyclohexanedione might have trapped the proposed sulfenic acid intermediate (**12**, Fig. 5). This conclusion was further substantiated by the studies using thio-ether **8** as the substrate under similar conditions. When thio-ether **8** was used as EgtE substrate, even in the presence of 50 × of 1,3-cyclohexanedione, ergothioneine was still the only detectable product (Supplementary Fig. 16).

**Figure 6:**
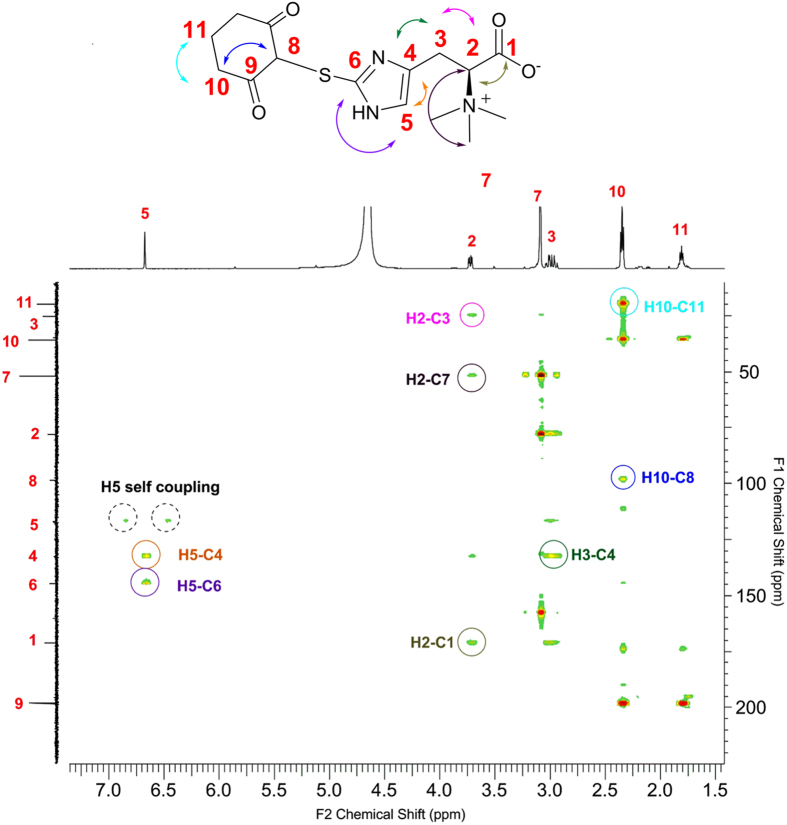
HMBC-NMR characterization of the sulfenic acid-dimedone adduct from the EgtE reaction.

To provide more evidence on the formation of an adduct between the proposed sulfenic acid intermediate **12** and 1,3-cyclohexanedione, the adduct was isolated and characterized. It has been reported that this type of adducts, the thio-ether derivative, was not stable under the acidic conditions^50^. Several attempted purifications using C_18_ HPLC methods failed due to the slight acidic environment during either the isolation or workup process. Later on, we discovered that the adduct can be isolated using cellulose resin under mild purification conditions. The adduct (**16**, Fig. 5) was fully characterized by ^1^H-NMR, ^13^C-NMR, 2-D NMR, and high-resolution mass spectrometry (Fig. 6 and Supplementary Fig. 17-21). ^1^H-^13^C correlations between H-2 and C-1, 3, 7 and ^1^H-^13^C correlations between H-5 and C-4, 6 in HMBC characterization supports compound **16** structural assignment (Fig. 6). Additional correlation between H-10 and C-8, 9 further supports the 1,3-cyclohexanedione portion in the compound **16**. Moreover, the ratios for the integration of each peaks from ^1^H-NMR spectrum (Supplementary Fig. 17) and the high-resolution mass spectrometry data (*m/z* [*M* + H]^+^ found 340.1342, calcd. 340.1331, Supplementary Fig. 19) were also consistent with the assignment of compound **16** structure.

In EgtE-catalysis, besides the production of ergothioneine, pyruvate and ammonia are the possible side-products. The identities of these two products were confirmed using three more assays: ^13^C-NMR analysis, ^1^H-NMR analysis after the 4-fluorophenylhydrazine treatment, and colorimetric characterization after treating the reaction mixture with Nessler′s reagent (K_2_HgI_4_ in KOH solution). From [β-^13^C]-labeled cysteine and hercynine, we synthesized [^13^C]-labeled sulfoxide 4 using the fungal paralog of EgtB (Egt1 from *N. crassa*, Supplementary Fig. 22)^21^ ^13^C-NMR analysis of EgtE catalysis is consistent with the production of pyruvate (Supplementary Fig. 23). The production of pyruvate was further validated after it was derivatized using fluorophenylhydrazine. After the EgtE reaction was quenched at 50 °C for 15 min, fluorophenylhydrazine was added into the reaction mixture and incubated at 50 °C for 3 h. Upon this treatment, pyruvate couples with 4-fluorophenylhydrazine to form an adduct (Supplementary Fig. 24)^51^, which allows us to quantify the ratio between ergothioneine and pyruvate from EgtE reaction mixture using ^1^H-NMR directly. Consistent with the proposed EgtE function, pyruvate and ergothioneine are produced at a ratio of ~1:1 in EgtE-catalysis (Supplementary Fig. 24). To verify the production of ammonia as the other product, Nessler′s reagent (K_2_HgI_4_ in KOH solution) was added to the mixture. The formation of the brown-orange color supports the production of ammonia in EgtE catalysis.^35^ The amount of ammonia was quantified based on the literature methods^34^,^35^. Results from this analysis also indicated that the ratio between ammonia to ergothioneine was ~1:1 (Supplementary Fig. 25). It can be concluded that ergothioneine, pyruvate and ammonia are the products of the EgtE-catalyzed C-S lyase reaction and these three products were produced at a ratio of ~1:1:1.

After the EgtE-catalytic system was established, EgtE kinetics was measured by monitoring the pyruvate formation rate. A colorimetric assay was developed by coupling the EgtE catalysis with the lactate dehydrogenase reaction^30^. In this assay, pyruvate produced from EgtE-catalysis was reduced to lactate by NADH (Supplementary Fig. 26) and the reaction rate was then measured by monitoring the NADH consumption rate at 340 nm. When sulfoxide **4** was used as substrate, the kinetic parameters were: *k*_cat_ = 1516 ± 27 min^−1^ and *K*_m_ = 121.2 ± 6.6 μM for compound **4**. When thio-ether **8** was used as substrate, the kinetic parameters are: *k*_cat_ = 476 ± 3 min^−1^ and *K*_m_ = 1960 ± 250 μM for compound **8**. In addition, it was discovered that the kinetic parameters are independent of DTT concentration whether sulfoxide **4** or thio-ether **8** was used as substrate. A difference of *k*_obs_/*K*_m_ of ∼ 52-folds for sulfoxide **4** relative to thio-ether **8** suggests sulfoxide **4** is the biological EgtE substrate.

## Discussion

Sulfur-containing molecules are widely distributed in nature, including amino acids, enzyme cofactors, antioxidants, nucleotides, and secondary metabolites^1^,^2^,^3^,^52^,^53^,^54^,^55^,^56^,^57^,^58^,^59^,^60^,^61^,^62^,^63^,^64^,^65^,^66^. Biological C-S bond formation and sulfur transfer reactions involve many novel transformations using either radical or ionic mechanisms. Biotin synthase and lipoate synthase-catalyzed sulfur insertions are anaerobic radical type chemistries. They belong to the radical SAM (*S*-adenosylmethionine) enzyme superfamily^54^,^67^,^68^,^69^,^70^,^71^, in which an iron-sulfur cluster is proposed to be the sulfur source. For ionic type of sulfur-transfer reactions, two types of activated sulfur species were reported: persulfide (R-S-SH) and thiocarboxylate (R-CO-SH)^59^. Persulfides can be either electrophiles or nucleophiles. The thiocarboxylate intermediates are normally located at the *C*-terminal of a protein before the sulfur is transferred to its target molecule^72^,^73^,^74^. The sulfur transfer strategy in ergothioneine biosynthesis is completely different from the previous literature examples and represents a novel sulfur transfer mechanism in synthesizing thio-containing natural products.

**Figure 7:**
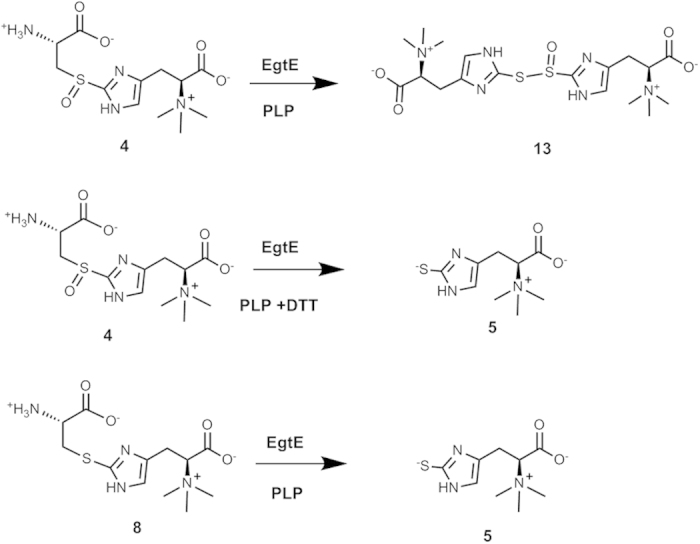
EgtE reactions under different *in vitro* conditions.

In ergothioneine biosynthesis, the net-transfer of sulfur from cysteine to histidine side-chain is the combination of two reactions: a mononuclear non-heme iron catalyzed oxidative C-S bond formation (EgtB or Egt1 catalysis, Fig. 1) and a PLP-dependent C-S lyase (EgtE). Due to the lack of access to *M. smegmatis* EgtE enzyme, the proposed C-S lyase activity in *M. smegmatis* was not reconstituted *in vitro. S*tudies were conducted using *M. smegmatis* cell lysate^36^. In this work, we have successfully produced EgtE protein and fully developed its catalytic system. Thus, for the first time, the ergothioneine biosynthetic pathway from *M. smegmatis* was fully reconstituted *in vitro*. EgtE enzymatic activities were characterized *in vitro* under a few different conditions: the C-S lyase activity for thio-ether **8** and the C-S lyase activities for sulfoxide **4** with and without reductants (Fig. 7). In all three reactions, pyruvate and ammonia were produced as the side-products. When thio-ether **8** was used as substrate, ergothioneine was the end product whether a reductant is present or not. However, when sulfoxide **4** was used as the substrate and in the absence of a reductant, thiol ester of thio-sulfinic acid (**13**) was the end product. Its presence was supported by the isolation of its hydrolyzed products: ergothioneine (**5**) and ergothioneine-2-sulfinic acid (**14**). When sulfoxide **4** was used as substrate and by including DTT as a reductant in the reaction mixture, only ergothioneine was produced. In the subsequent characterizations of EgtE catalysis using a coupled assay to monitor pyruvate formation rate, the kinetic parameters suggest that the sulfoxide **4** is the preferred substrate for EgtE enzyme. This is also consistent with previous studies on the mononuclear non-heme iron enzyme (Egt1, EgtB, or OvoA) catalyzed oxidative C-S bond formation reactions (Fig. 1)^20^,^21^,^37^,^38^,^75^. Several lines of evidence suggest that sulfoxide **4** instead of a thio-ether **8** is the oxidative coupling product from these mononuclear non-heme iron enzyme catalyzed reactions: 1) H_2_O_2_ was not detected as a side-product in this reaction; 2) Thio-ether **8** was synthesized chemically and under the conditions, its oxidation to sulfoxide **4** by either O_2_ or H_2_O_2_ is below our detection limit; 3) When 40 × of catalase relative to OvoA, Egt1 was included in the reaction mixture, sulfoxide **4** was still the only detectable oxidative coupling product^75^. Thus, detailed biochemical characterizations of the two key enzymes (EgtB/Egt1 and EgtE) led to the full reconstitution of the ergothioneine biosynthetic pathway *in vitro* (Fig. 1).

**Figure 8:**
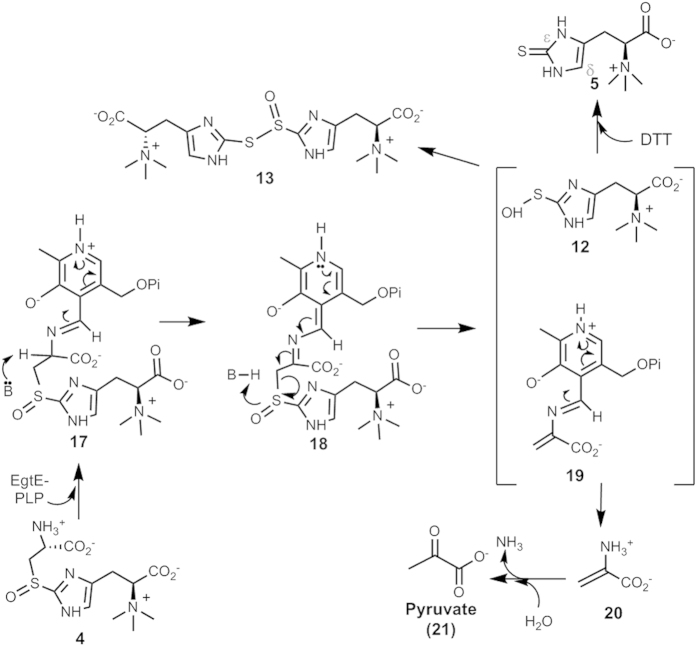
Proposed EgtE mechanistic model.

EgtB-catalysis seems to be distinct from all currently known C-S bond formation chemistries. EgtB is a mononuclear non-heme iron enzyme and it was proposed that Fe^IV^ = O species are involved in this oxidative C-S bond formation process^37^,^38^,^76^,^77^. The proposed mechanistic models still await to be verified by future trapping and characterization of the proposed intermediates. Besides the novel C-S bond formation chemistry catalyzed by EgtB, the EgtE catalysis is equally intriguing. EgtE is a PLP-containing enzyme based on both bioinformatic sequence analysis and characterization of the purified protein. To explain all of the discoveries reported in this study, we proposed an EgtE mechanistic model (Fig. 8). Similar to other PLP-containing C-S lyases^34^,^35^, the first step is the formation of the Schiff-base (**17**) between the PLP cofactor and the substrate (**4**), deprotonation of the Cys α-carbon leads to intermediate **18**. The subsequent C-S bond cleavage produces a PLP-based adduct (**19**) and a sulfenic intermediate of hercynine (**12**), which is released from the active site into the solvent environment. Due to its instability, the disproportionation reaction between two molecules of **12** will lead to the formation of a thiol ester of thio-sulfinic acid **13**, which is the compound detected in EgtE reaction when a reductant was not included in the reaction mixture (Fig. 4D). In the presence of DTT, ergothioneine sulfenic acid (**12**) will be reduced to ergothioneine (Fig. 4E). In the C-S lyase reaction of intermediate **18**, besides sulfenic acid **13**, a PLP-based intermediate **19** will also form. Similar to other C-S lyases^35^, the amine exchange between this intermediate and an EgtE active site lysine residue leads to the production of pyruvate (**21**) and ammonia as the side-products. This mechanistic model is consistent with the production of ergothioneine, pyruvate, and ammonia in a ratio of 1:1:1 based on the quantitative analysis of these three products. This model can also be used to explain the result when thio-ether **8** was used as substrate. When thio-ether **8** is used as substrate, the C-S lyase reaction from an intermediate analogous to intermediate **18** will produce ergothioneine directly. Thus, when thio-ether **8** is used as substrate, no reduction is required. In addition, the successful isolation of the adduct (**16**) between the proposed sulfenic acid intermediate **12** and dimedone provided further evidence supporting the proposed EgtE reaction mechanism. Our EgtE product profile is also consistent with the behaviors of ergothioneine under oxidative conditions, in which sulfenic acid intermediate was also suggested to be involved^42^. The successful trapping and characterization of sulfenic acid intermediate in EgtE catalysis provide evidences supporting the involvement of ergothioneine sulfenic acid as a key intermediate when ergothioneine is exposed to oxidative conditions as suggested by Servillo *et al.*^42^

## Methods

### General methods

The cloning, expression and purification of the EgtE protein are described in the Supplementary Methods.

### The pH-dependence of the EgtE UV-vis spectra

The universal buffer containing 25 mM NaOAc, 25 mM MES, 25 mM glycine, 75 mM Tris was prepared as 6.0, 7.0, 8.0, 9.0, 10.0. EgtE (400 μM) was diluted to 30 μM with universal buffer and UV spectrums were recorded at each pH.

### PLP content determination

The cofactor (PLP) content was determined by adding NaOH to purified EgtE solution to a final concentration of 0.2 M to denature the protein and release the tightly bound PLP. The absorbance at 388 nm was used to determine the concentration of the cofactor, which in 0.2 M NaOH exhibits a molar extinction coefficient of 6600 M^−1^ cm^−1^ 32. The concentration of EgtE was determined by amino acid analysis. The PLP content was calculated based on EgtE and PLP concentrations.

### Synthesis of the thio-ether substrate (8)

Thio-ether **8** was prepared following a literature procedure^39^. Hercynine (56 mg) was dissolved in 3 mL H_2_O on ice and 1.5 equivalents of concentrated HCl was added. A volume of 45 μL of Br_2_ was added drop-wise to the reaction mixture. After stirring for 7 min, 168 mg cysteine (5 equivalents) was added into the reaction mixture and the reaction was stirred on ice for an additional hour. The white precipitate was filtered and the supernatant was loaded on SCX resin (H^+ ^form), washed with water and 1N HCl. Thio-ether **8** was eluted with 1N NH_4_OH (aqueous) and was analyzed using Varian 500 MHz ^1^H-NMR spectroscopies, and high-resolution mass spectrometry.

^**1**^**H-NMR** (500MHz, 20 °C) of **8**: δ 3.02-3.24 (m, 13H), δ 3.32 (dd, J = 3.9, 14.7 Hz, 1H), δ 3.64 (dd, J = 3.9, 7.3 Hz, 1H), δ 3.74 (dd, J = 3.9, 11.7, 1H), δ 6.90 (s, 1 H);

^**13**^**C-NMR** (125 MHz, 20 °C) of **8**: δ 23.30 (3), 36.34 (3′), 52.05 (2′), 53.86 (7′), 76.12 (2), 121.49 (5), 130.01 (4), 139.06 (6), 169.10 (1′), 171.08 (1). Please refer the structure in Supplementary Fig. 3 for the numbering system;

**High resolution ESI-MS** of **8**: Calculated molecular weight for compound **8** as [M-2H]^–^ (negative mode) form is 315.1138, and found 315.1124.

### Isolation and characterization of ergothioneine 5 from the EgtE reaction

A reaction assay mixture in a 10 mL of final volume contained 50 mM KPi buffer, pH 8.0, 2 mM compound **4**, 2 mM DTT and 1.0 μM EgtE protein. The mixture was incubated at 28 °C for 1 hour. The EgtE protein in the reaction mixture was removed by ultra-filtration. The desired product was purified by HPLC (C_18_ reversed phase column, waters, 250 × 10 mm, mobile phase: 1 mL/min flow of H_2_O containing 2% acetonitrile). Purified ergothioneine **5** was lyophilized and dissolved in 400 μL of D_2_O. Ergothioneine **5** was analyzed using Varian 500 MHz ^1^H-NMR spectroscopies, and high-resolution mass spectrometry.

^**1**^**H-NMR** (500 MHz, 20 °C) of ergothioneine **5**: δ 2.99-3.16 (m, 11H), δ 3.73 (dd, J = 3.9, 11.7 Hz, 1H), δ 6.64 (s, 1 H);

^**13**^**C-NMR** (125 MHz, 20 °C) of ergothioneine **5**: δ 23.12 (3), 52.35 (7), 76.99 (2), 115.22 (5), 123.78 (4), 155.80 (6), 170.22(1); Please refer the structure in Supplementary Fig. 7 for the numbering system;

**High-resolution ESI-MS** of ergothioneine **5**: Calculated molecular weight for ergothioneine **5** as [M-2H]^–^ (negative mode) form is 228.0812, and found 228.0820.

### NMR characterizations of the EgtE reaction mixture in the absence of reductants

A reaction assay mixture in a 10 mL of final total volume contained 50 mM KPi buffer, pH 8.0, 2.0 mM of sulfoxide **4**, 2.0 mM DTT and 1.0 μM EgtE protein. The mixtures were incubated at 28 °C for 1 hour. The EgtE protein in the reaction mixture was removed by ultra-filtration. The desired product was purified by HPLC (C18 reversed phase column, waters, 250 × 10 mm, mobile phase: 1 mL/min flow of H_2_O containing 2% acetonitrile). Purified compounds **5** and **14** was lyophilized and dissolved in 400 μL of D_2_O. Compound **14** was analyzed using Varian 500 MHz ^1^H-NMR spectroscopies, and high-resolution mass spectrometry.

^**1**^**H-NMR** (500 MHz, 20 °C) of **14**: δ 2.99-3.16 (m, 11H), δ 3.73 (dd, J = 3.9, 11.7 Hz, 1H), δ 6.64 (s, 1 H);

^**13**^**C-NMR** (125 MHz, 20 °C) of **14**: δ 22.73 (3), 52.05 (7), 75.08 (2), 120.22 (5), 130.78 (4), 143.80 (6), 169.98(1); Please refer the structure in Supplementary Fig. 11 for the numbering system;

**High-resolution ESI-MS** of **14**: Calculated molecular weight for **14** as [M-H]^+^ (positive mode) form is 262.0812, and was found 262.1035.

### Trapping the sulfenic acid intermediate in EgtE reaction

EgtE reaction in the presence of 50 × cyclohexane-1,3-dione relative to sulfoxide **4** was performed to trap the proposed sulfur sulfenic acid intermediate (**12**). The reaction contained 3.5 mM sulfoxide substrate **4**, 165 mM cyclohexane-1,3-dione in 100 mM KPi buffer, pH 8.0, was initiated by the addition EgtE to a final concentration of 4.0 μM (A). EgtE reaction using thio-ether **8** as substrate in the presence of 50 × cyclohexane-1,3-dione was conducted as a control experiment (B). EgtE reaction with sulfoxide substrate **4** as substrate in the absence of cyclohexane-1,3-dione was also conducted under identical conditions as an additional control (C).

### Isolation of the trapped intermediate

The cellulose resin was packed using iPrOH: ACN = 4.5 : 2.5 into the column (1 × 20 cm). The EgtE reaction, in the presence of 50 × of dimedone relative to sulfoxide **4** concentration, was lyophilized and the resulting powder was dissolved in 0.5 mL of iPrOH: ACN = 4.5 : 2.5. The mixture was loaded onto the column and washed with 16 × 5.0 mL of iPrOH: ACN = 4.5 : 2.5. The desired compound was then eluted using the solvent system of iPrOH: ACN: 0.1 M NH_4_HCO_3_ = 4.5 : 2.5 : 3. The fractions contained target compounds were collected and the solvents were removed by rotary evaporation. Purified compound **16** was lyophilized and dissolved in 400 μL of D_2_O and characterized using Varian 500 MHz ^1^H-NMR spectroscopies, and high-resolution mass spectrometry.

^**1**^**H-NMR** (500 MHz, 20 °C) of **16**: δ 1.81 (dt, J = 6.4, 12.7, 2H), δ 2.35 (t, J = 6.4, 4H), δ 2.96 (dd, J = , 12.7, 2H), δ 3.02 (dd, J = 3.9, 11.7 Hz, 1H), δ 3.09 (s, 9H), δ 3.72 (dd, J = 4.4, 10.8 Hz, 1H), δ 3.73 (dd, J = 3.9, 11.7 Hz, 1H), δ 6.67 (s, 1 H);

^**13**^**C-NMR** (125 MHz, 20 °C) of **16**: δ 20.05 (11), 25.38(3), 36.04(10), 52.01 (7), 78.19 (8), 98.38 (2), 117.02 (5), 132.80 (4), 144.34(6), 171.03 (1), 198.15(9); Please refer the structure in Supplementary Fig. 18 for the numbering system;

**High resolution ESI-MS** of **16**: Calculated molecular weight for compound **16** as [M-H]^+^ (positive mode) form is 340.1331, and was found at 340.1342.

### Quantification of NH_4_^+^ produced in EgtE reaction

NH_3_ produced from EgtE reaction will be protonated to the form NH_4_^+^. Therefore, to accurately quantify the amount of NH_3_ formed in EgtE reaction, NH_4_^+^ ions present in the substrate has to be removed first. NH_4_^+^ could be converted to NH_3_ gas by adjusting the pH to basic. To remove NH_4_^+^ from the substrate, EgtE substrate (24 μL, 27 mM) was diluted into 100 μL of H_2_O. The pH was adjusted to 13.0 by NaOH (aqueous). The sample was frozen and lyophilized to dryness. The resulting powder was re-dissolved into 100 μL water and lyophilized again. This process was repeated three times in total. The final powder was dissolved in 100 μL H_2_O and adjusted to neutral using concentrated HCl (aqueous). The substrate solution (200 μL) in neutral condition was divided into two even portions: one for EgtE reaction and the other for control experiment. The EgtE reaction (200 μL) mixture contained 5.0 μM EgtE, 1.62 mM substrate, 2 mM DTT in 50 mM KPi buffer, pH = 8.0. The control reaction contained the same components except that denatured EgtE was used to replace native EgtE. Both reactions were incubated at 30 °C for one hour. To the reaction mixture, H_2_O (700 μL) and Nessler’s reagent (100 μL) were added to make a final solution of 1 mL. The control reaction was used for deduction of any residual NH_4_^+^ in substrate solution. Absorption was measured at 462 nm. The reaction sample provided the reading of 0.7881 ± 0.0035. At the same time, a standard curve was generated using NH_4_^+^ solutions and used to calculate the amount of NH_4_^+^ produced from the EgtE reaction. Based on our measurement, the ratio between formed NH_4_^+^ and the ergothioneine is ∼1:1.

### Determining the production of pyruvate as the other side-product

It was difficult to directly measure the ratio between ergothioneine and pyruvate from ^1^H-NMR since the methyl hydrogen of pyruvate is solvent exchangeable in deuterated NMR solvents. To avoid this issue, 4-fluorophenylhydrazine was added into the reaction mixture to quantitatively convert pyruvate to 2-(2-(4-fluorophenyl)hydrazono)propanoate (**23**) and then used for quantification using ^1^H-NMR method (Supplementary Fig. 27).^51^ EgtE reaction contained 1.0 mM substrate, 1.0 mM DTT and 4.0 μM EgtE in 50 mM KPi buffer, pH 8.0. The reaction was incubated at 28 °C for 0.5 h. After EgtE reaction was complete, 2.0 mM 4-fluorophenylhydrazine was added to the reaction mixture and incubated at 50 °C for 3 hours. The reaction mixture was lyophilized and analyzed by ^1^H-NMR. Based on NMR characterization of 2-(2-(4-fluorophenyl)hydrazono)propanoate (**23**), chemical shifts for methyl hydrogen are: δ 1.91 (s, 3H) and ergothioneine imidazole hydrogen chemical shift is 6.65 ppm. Direct quantification of ergothioneine imidazole hydrogen and methyl hydrogen of 2-(2-(4-fluorophenyl)hydrazono)propanoate (**23**) in ^1^H-NMR spectra would lead to an inaccurate integration because their signals are far from each other and are located on the two sides of the large water signal. To avoid this issue, ethyl viologen (as shown in Supplementary Fig. 24) was used as an internal standard to calibrate the ratio of 2-(2-(4-fluorophenyl)hydrazono)propanoate (**23**) and ergothioneine because ethyl viologen has signals at both low field and high field ranges (please refer to Supplementary Fig. 24 for the compound numbering system). Ethyl viologen chemical shifts used in this analysis are: δ 1.56 (t, 7.3 Hz, 6H), δ 8.40 (d, 6.4 Hz, 4H), and δ 8.99(d, 6.4 Hz, 4H). Once the ratio between the ergothioneine and ethyl viologen and the ratio between 2-(2-(4-fluorophenyl)hydrazono)propanoate (**23**) and ethyl viologen were determined, the ratio between ergothioneine and 2-(2-(4-fluorophenyl)hydrazono)propanoate (**23**) was calculated by dividing these two ratios. Using this method, the ratio between 2-(2-(4-fluorophenyl)hydrazono)propanoate (**23**) and ergothioneine was determined to be ~1:1.

### EgtE kinetic characterization by lactate dehydrogenase-coupled assay

Because pyruvate is produced as one of the side-products in EgtE reaction, the EgtE kinetics was measured by coupling the EgtE reaction with the lactate dehydrogenase and monitoring the NADH consumption rate. A typical assay mixture contained 10 nM of EgtE, 0.13 mM NADH, 1.0 mM DTT, 22.5 U/mL LDH (2000 × relative to EgtE activity used in the assay) in 50 mM KPi buffer, pH 8.0 and various amounts of EgtE substrate in a total volume of 1.0 mL. The reaction was monitored at 340 nm using the Varian Cary 100 Bio UV-vis spectrometer. The data was fitted by SigmaPlot.

## Additional Information

**How to cite this article**: Song, H. *et al.* Mechanistic studies of a novel C-S lyase in ergothioneine biosynthesis: the involvement of a sulfenic acid intermediate. *Sci. Rep.* **5**, 11870; doi: 10.1038/srep11870 (2015).

## Supplementary Material

Supplementary Information
