# Supplementary material for: Mechanistic studies of a novel C-S lyase in ergothioneine biosynthesis: the involvement of a sulfenic acid intermediate
Source: Sci Rep. 2015 Jul 7;5:11870. doi: 10.1038/srep11870 (PMC4493562; doi:10.1038/srep11870)
Supplement: Supplementary Information [file srep11870-s1.pdf]

Supporting Information

Mechanistic studies of a novel C-S lyase in ergothioneine biosynthesis: the involvement of a sulfenic acid intermediate

Heng Song,[1†] Wen Hu,[1,2†] Nathchar Naowarojna,[1] Ampon Sae Her,[1] Shu Wang,[1] Rushil Desai,[1] Li Qin,[2*] Xiaoping Chen,[2*] and Pinghua Liu[1]*

[1]*Department of Chemistry, Boston University, 590 Commonwealth Ave, Boston, MA 02215, USA*

[2]*State Key Laboratory of Respiratory Disease, Guangzhou Institutes of Biomedicine and Health, Chinese Academy of Sciences, Guangzhou, 510530, P. R. China*

[#]*Contributed equally to the work.*

E-mail: pinghua@bu.edu

Table of Contents

Supporting information (protein purification, kinetic characterization, product isolation, and characterization by NMR and mass spectrometry):

A. The UV-visible spectrum and SDS-PAGE gel of the anaerobically purified EgtE;
B. Characterizations of the synthesized thio-ether substrate **8**;
C. NMR characterizations of the EgtE reaction mixture in the absence of reductants;
D. NMR characterizations of EgtE reaction products;
E. NMR characterizations of the sulfenic acid-dimedone adduct **(16)**;
F. Quantification of the ratio between ergothioneine and pyruvate in EgtE reaction;
G. Quantification of NH_4^+ produced in EgtE reaction;
H. EgtE steady-state kinetic analysis using sulfoxide substrate **4** as substrate;
I. DTT effect on EgtE activity using sulfoxide substrate **4** as substrate;
J. EgtE steady-state kinetic analysis using thioether substrate **8** as substrate.

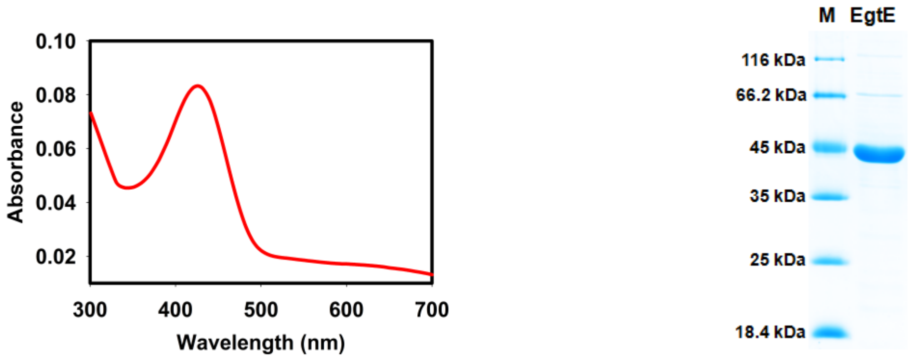

Supplementary Figure 1. 1) The UV-visible spectrum (pH 7.5) of the anaerobically purified EgtE using Strep-Tactin resin from IBA, Inc. 2) SDS-PAGE gel of the purified EgtE protein.

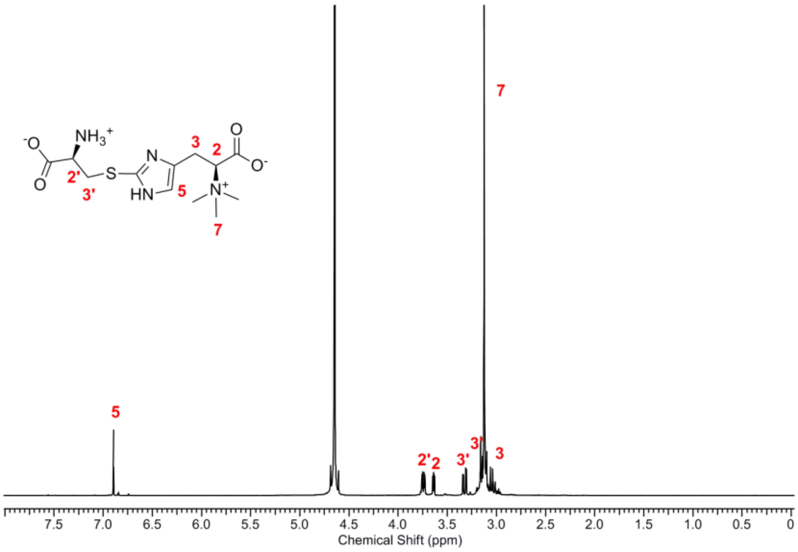

Supplementary Figure 2. ^{1}H-NMR spectrum of the thio-ether substrate **8**.

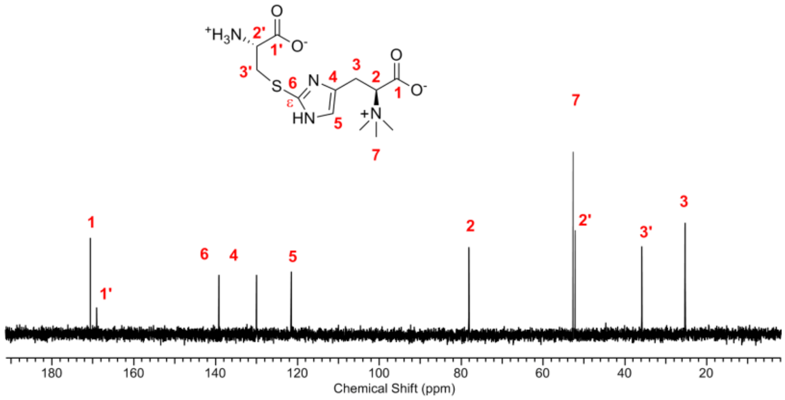

Supplementary Figure 3. ^{13}C-NMR spectrum of the thio-ether substrate **8**.

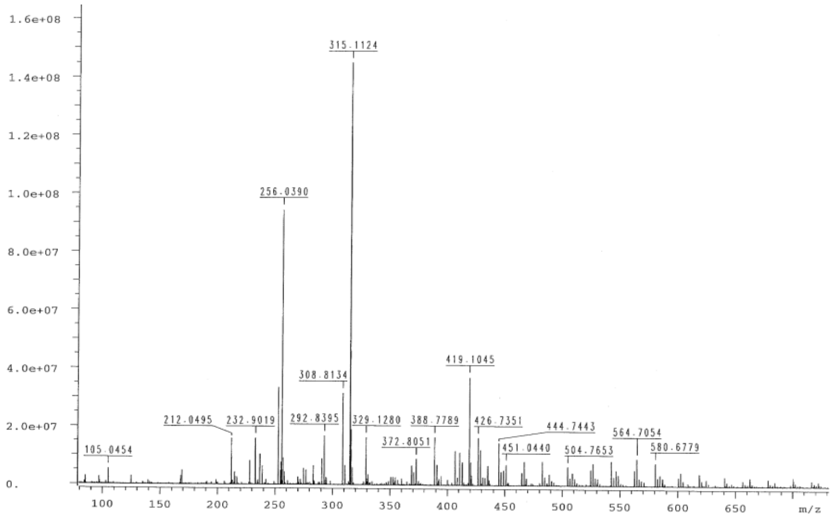

Supplementary Figure 4. High-resolution mass spectrometry spectrum of the thio-ether substrate **8**. (Detected at 315.1124, Calculated for $C_{12}H_{19}N_4O_4S^-$ at 315.1132).

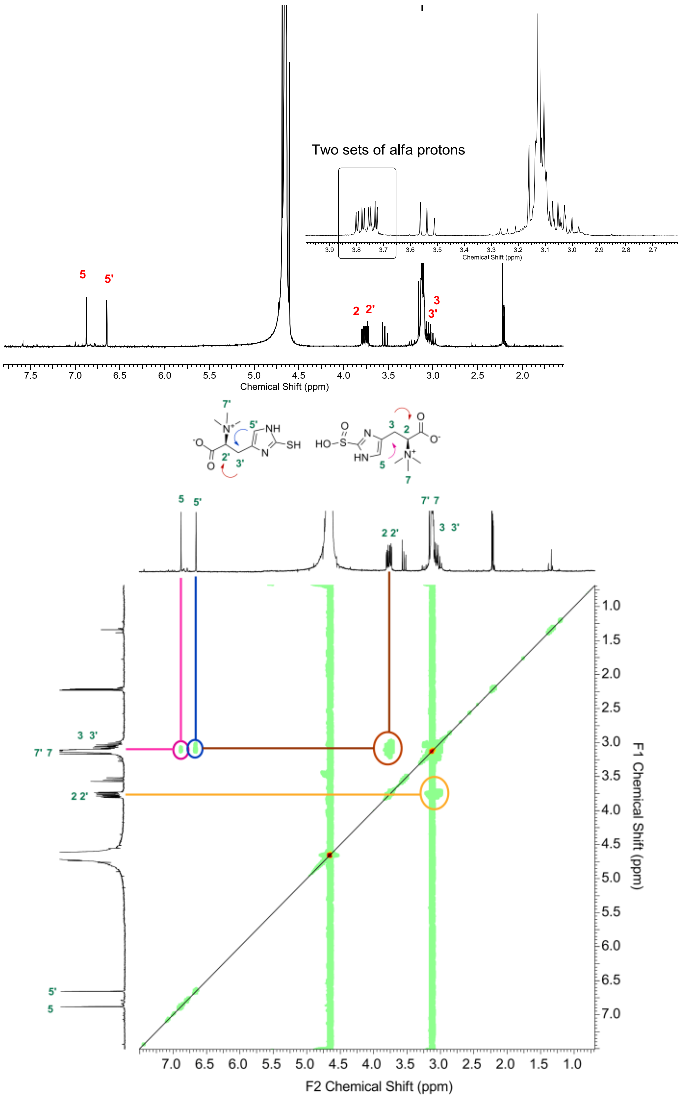

Supplementary Figure 5. ^{1}H-NMR spectrum (top) and 2D-gCOSY-NMR spectrum (bottom) of the EgtE reaction mixture in the absence of reductants.

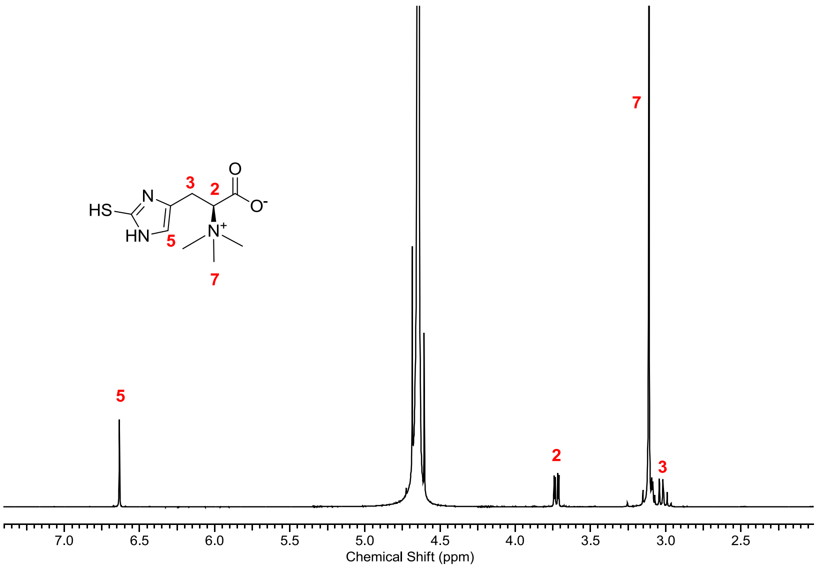

Supplementary Figure 6. ^{1}H-NMR spectrum of ergothioneine **5**.

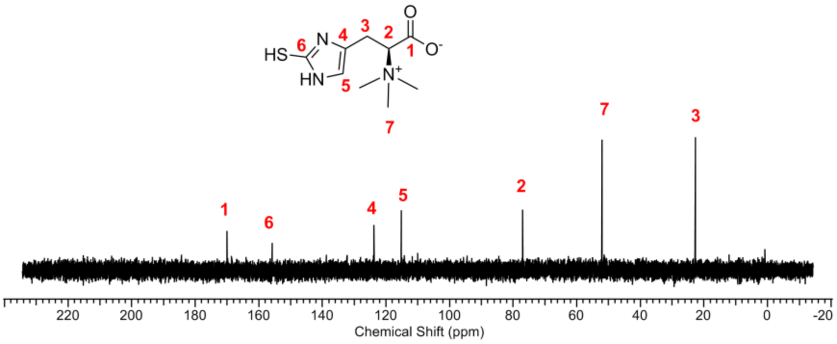

Supplementary Figure 7. ^{13}C-NMR spectrum of ergothioneine **5**.

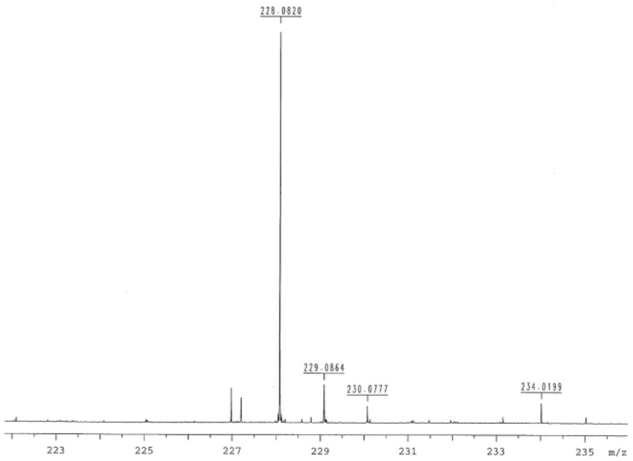

Supplementary Figure 8. High-resolution mass spectrometry spectrum of ergothioneine **5**. Calculated molecular weight for compound **5** as $[M-2H]^-$ (negative mode) form was 228.0812, and found 228.0820.

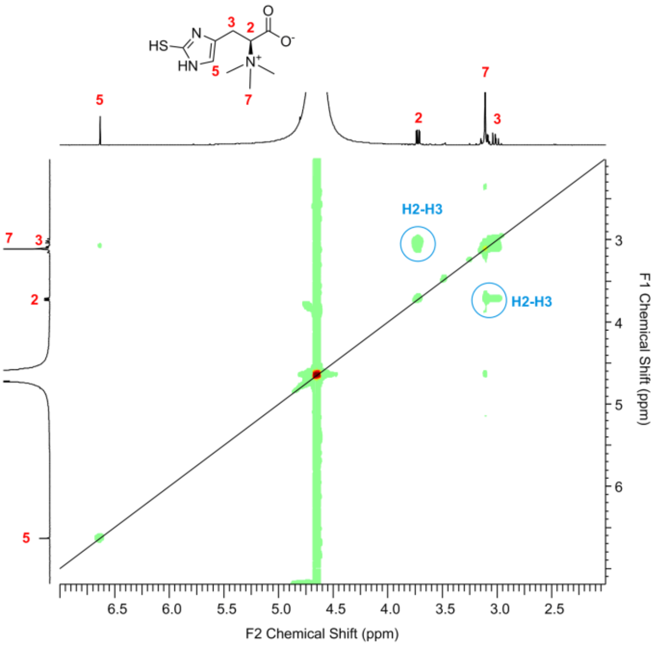

Supplementary Figure 9. COSY-NMR spectrum of ergothineine **5**.

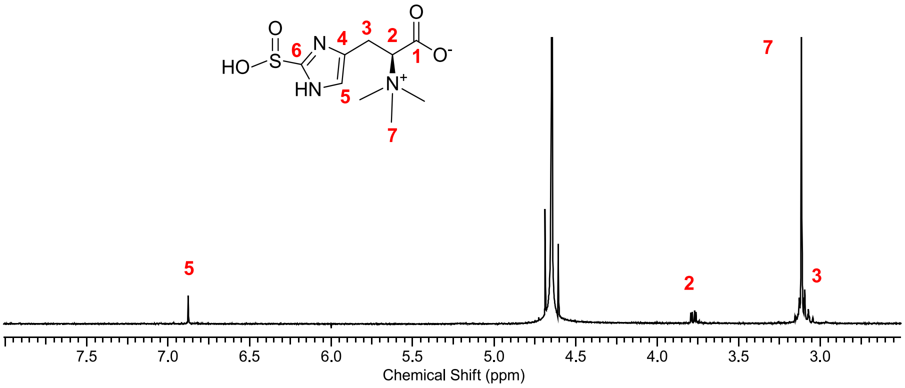

Supplementary Figure 10. ^{1}H-NMR spectrum of ergothineine-2-sulfinic acid **14**.

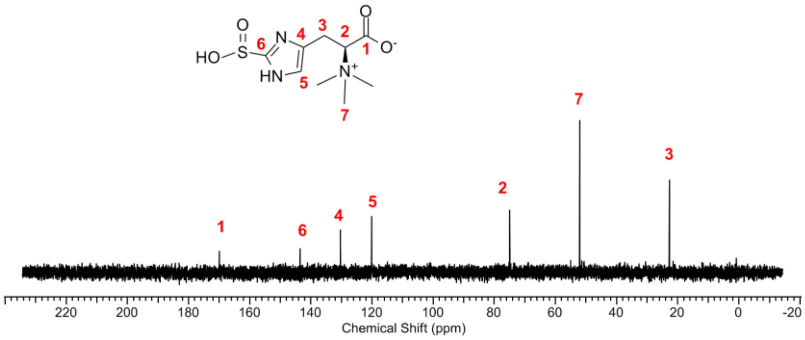

Supplementary Figure 11. ^{13}C-NMR spectrum of ergothineine-2-sulfinic acid **14**.

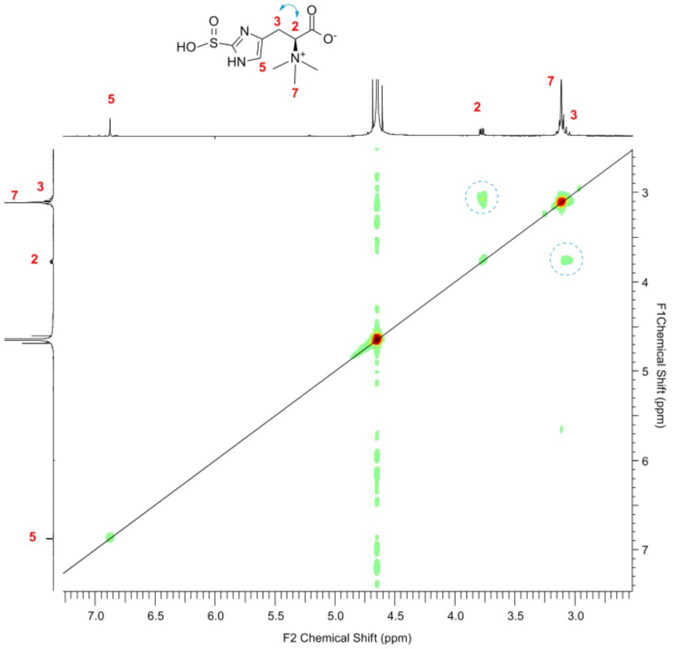

Supplementary Figure 12. COSY-NMR spectrum of ergothineine-2-sulfinic acid **14**.

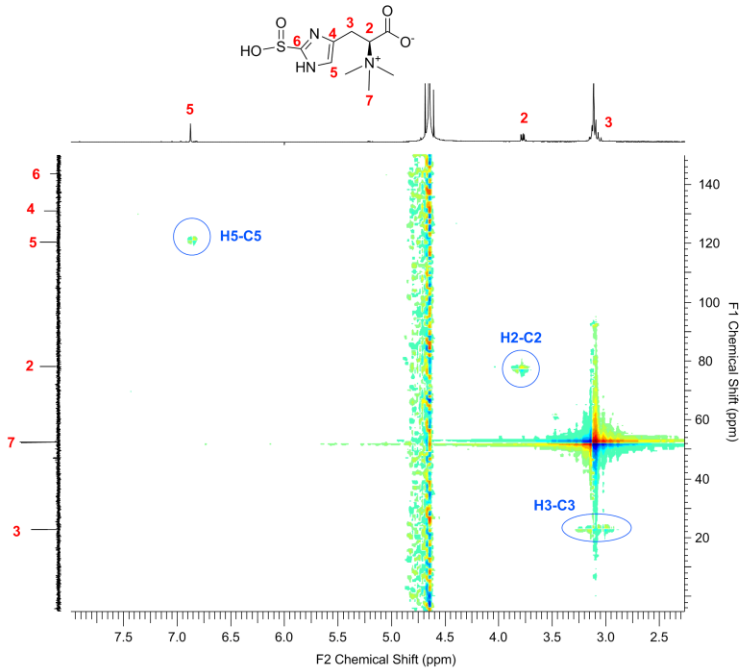

Supplementary Figure 13. HMQC-NMR spectrum of ergothineine-2-sulfinic acid **14**.

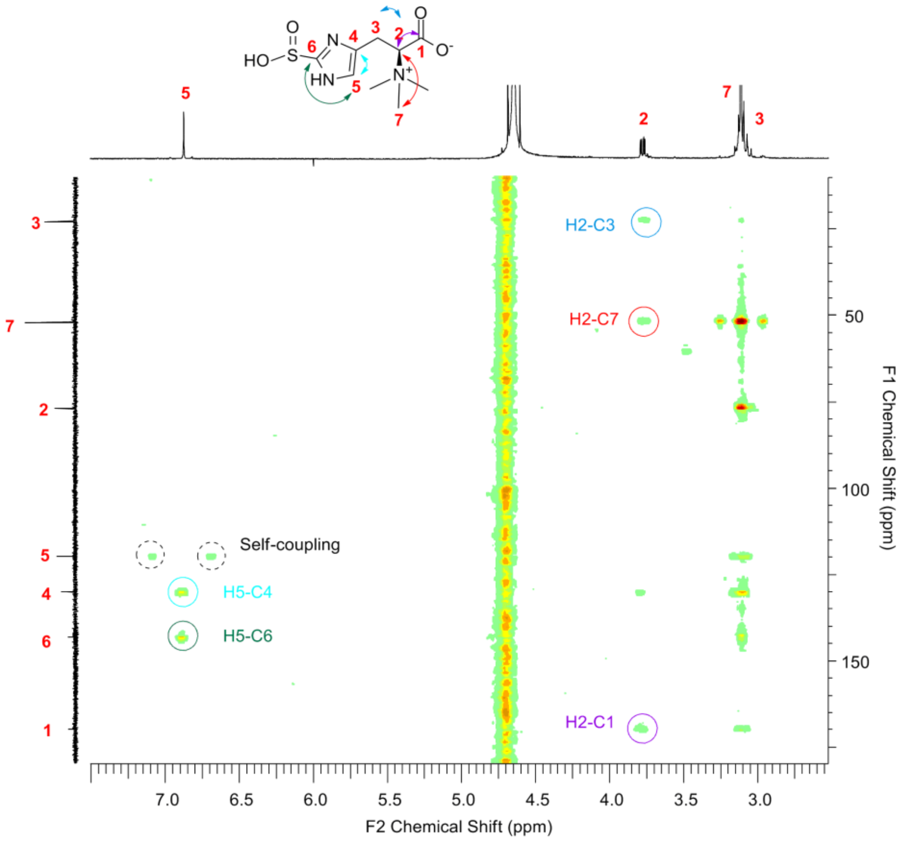

Supplementary Figure 14. HMBC-NMR spectrum of ergothineine-2-sulfinic acid **14**.

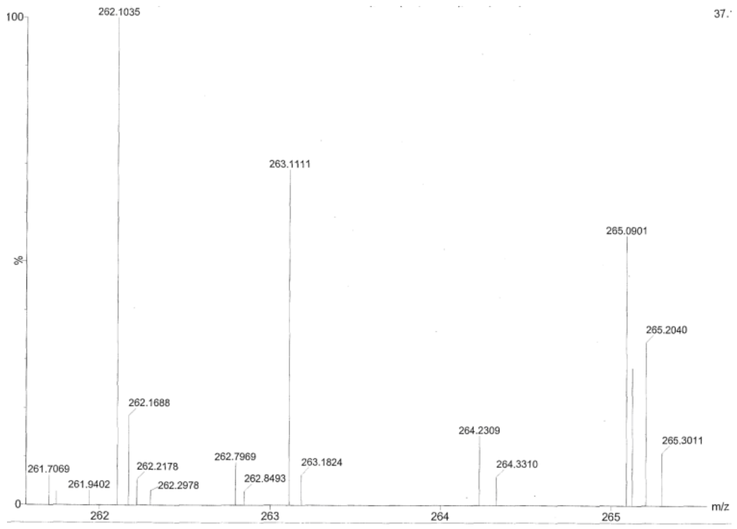

Supplementary Figure 15. HRMS spectrum of ergothineine-2-sulfinic acid **14**. Calculated molecular weight for compound **14** as [M-H]$^+$ (negative mode) form was 262.0812, and found 262.1035.

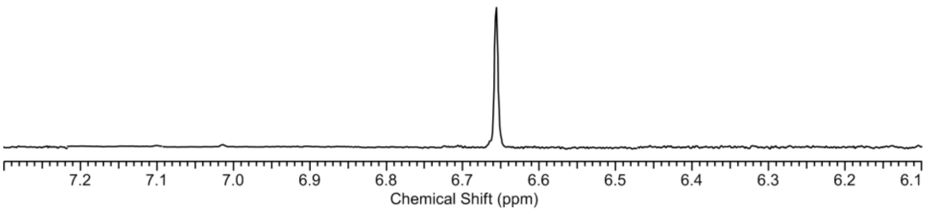

Supplementary Figure 16. ^{1}H-NMR spectrum of EgtE reactions using either thio-ether **8** as the substrate and in the presence of 50 × of 1,3-cyclohexanedione. Ergothioneine **5** is still the only detectable product.

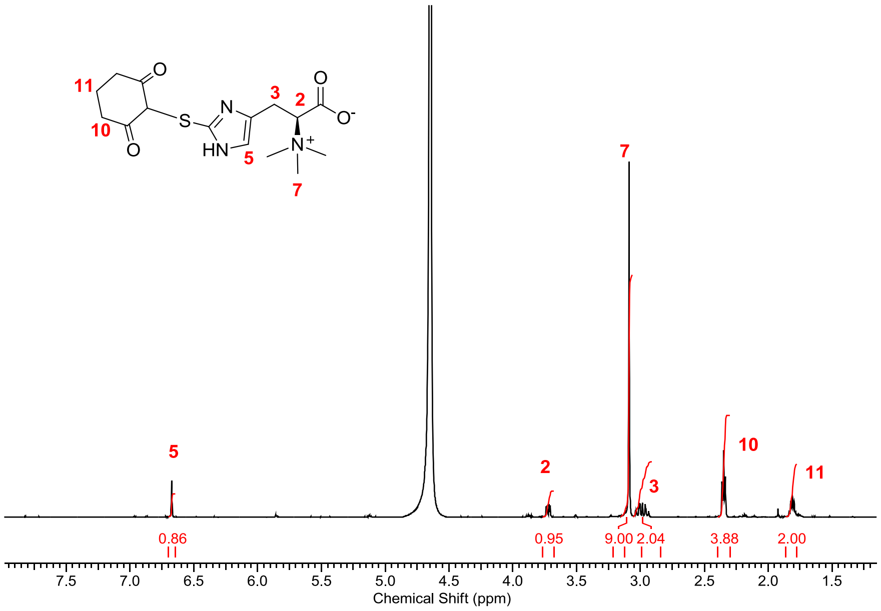

Supplementary Figure 17. ^{1}H-NMR of the isolated sulfenic acid-dimedone adduct **16** using cellulose chromatography.

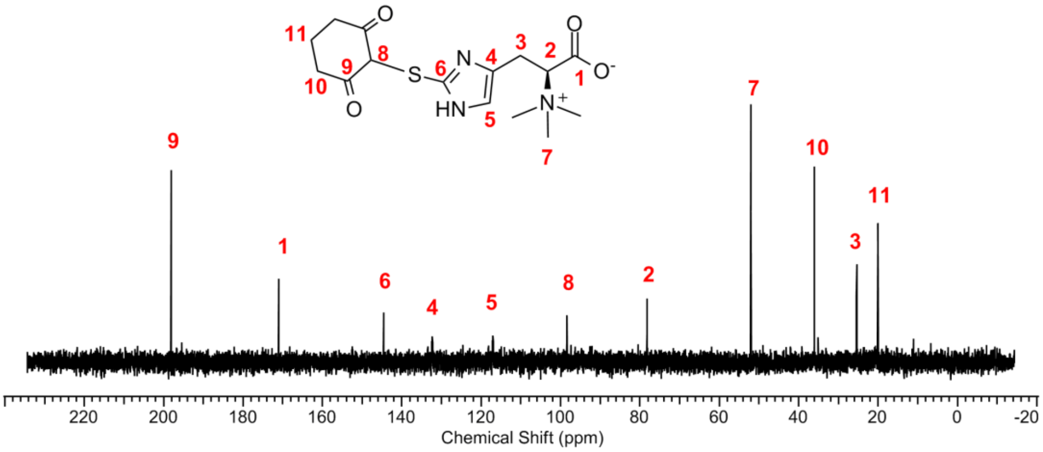

Supplementary Figure 18. ^{13}C-NMR of the isolated sulfenic acid-dimedone adduct **16** from cellulose purification.

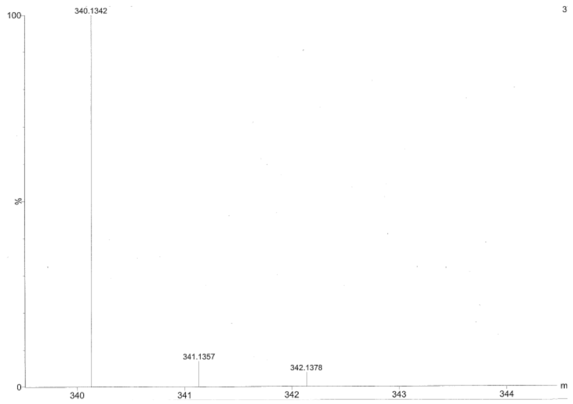

Supplementary Figure 19. HRMS of the isolated sulfenic acid-dimedone adduct **16** from cellulose chromatography. Calculated molecular weight for compound **16** as $[M-H]^+$ (positive mode) form was 340.1331, and found 340.1342.

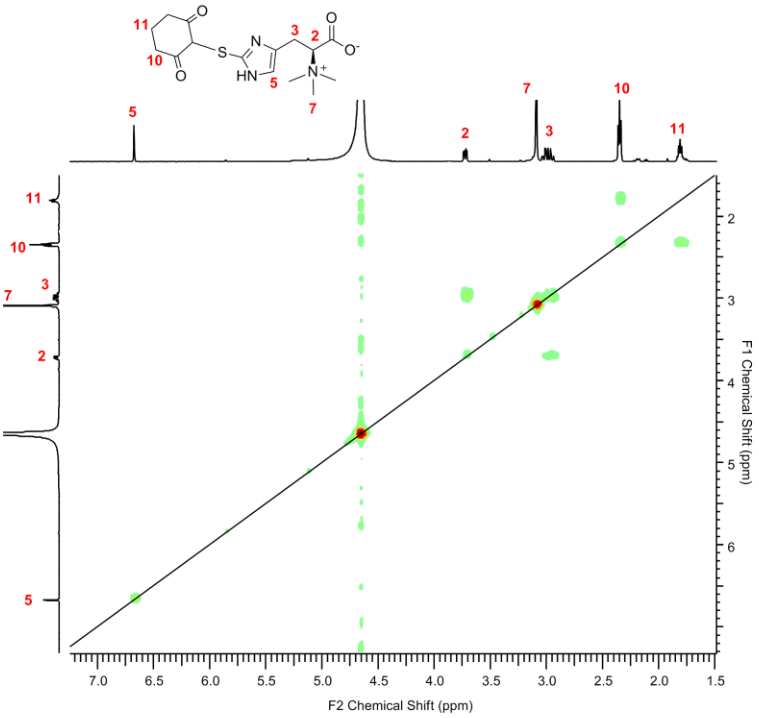

Supplementary Figure 20. COSY-NMR of the isolated sulfenic acid-dimedone adduct **16** from cellulose chromatography.

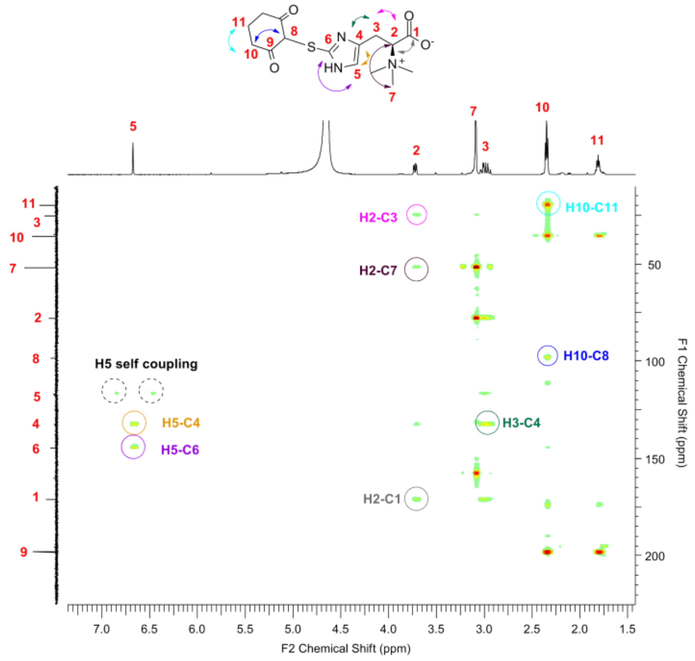

Supplementary Figure 21. HMBC-NMR of the isolated sulfenic acid-dimedone adduct **16** from cellulose chromatography.

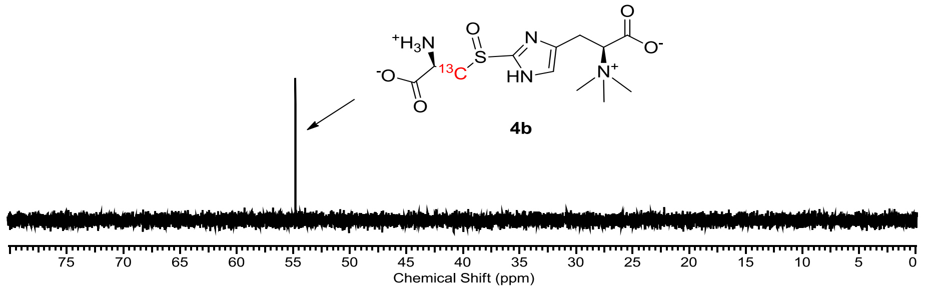

Supplementary Figure 22. ^{13}C-NMR spectrum of [^{13}C]-labeled sulfoxide substrate (**4b**) from *N. crassa* Egt1.[4]

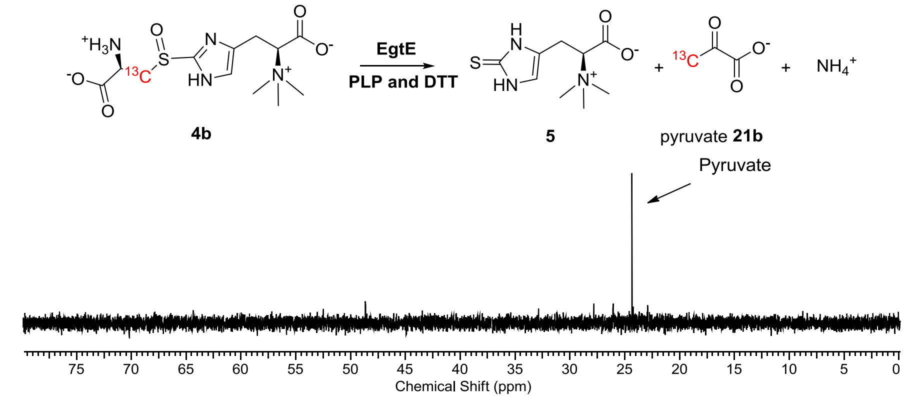

Supplementary Figure 23. ^{13}C-NMR spectrum of EgtE reaction using ^{13}C-labeled sulfoxide substrate (**4b**) in the presence of DTT.

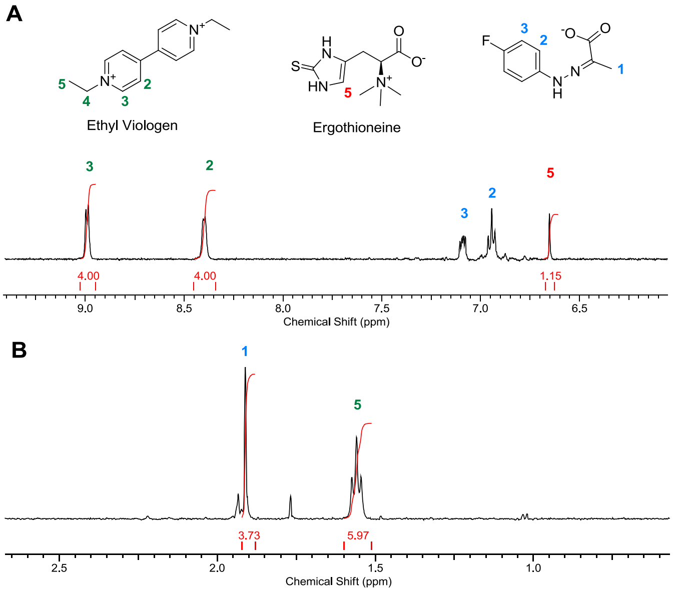

Supplementary Figure 24. ^{1}H-NMR spectrum of the EgtE-reaction for quantifying the ratio between ergothioneine and pyruvate. **A:** Expanded regions between 6.0 ppm - 9.5 ppm. **B:** Expanded regions between 0.5 - 2.7 ppm regions. After the EgtE reaction was quenched at 50 ˚C for 15min, fluorophenylhydrazine (**22**) was added into the reaction mixture and incubated at 50 °C for 3h. Upon this treatment, pyruvate couples with 4-fluorophenylhydrazine (**22**) to form an adduct (**23**) (Supplementary Figure 27). Upon treatment with fluorophenylhydrazine, the pyruvate methyl group changed from solvent-exchangeable to non-exchangeable, which allowed us to directly quantify the ratio between ergothioneine and pyruvate from EgtE reaction mixture using ^{1}H-NMR directly. To quantitatively measure the ratio between ergothioneine **5** and the adduct **23**, ethyl viologen was added as an internal standard to improve the accuracy of signal quantification. Ethyl viologen has two signals in the 8 – 9.5 ppm region (labeled as 2 and 3, colored green in the spectrum) and signals at ~ 1.5 ppm region (labeled as 5 and colored green in the spectrum). The ratio between ergothioneine **5** and the ethyl viologen signals was calculated by measuring the ratio between ergothioneine **5** H-5 signal (labeled as **5** and colored red in the spectrum) and the ethyl viologen aromatic hydrogen signals. The ratio between the adduct **23** and the ethyl viologen signals was calculated by measuring the ratio between methyl group of the adduct **23** (labeled as 1 and colored blue in the spectrum) and the ethyl viologen ethyl group hydrogen signals (labeled as 5 and colored green in the spectrum). Based on the integration using ethyl viologen as an internal standard, the ratio of ergothioneine and pyruvate was ~ 1: 1.

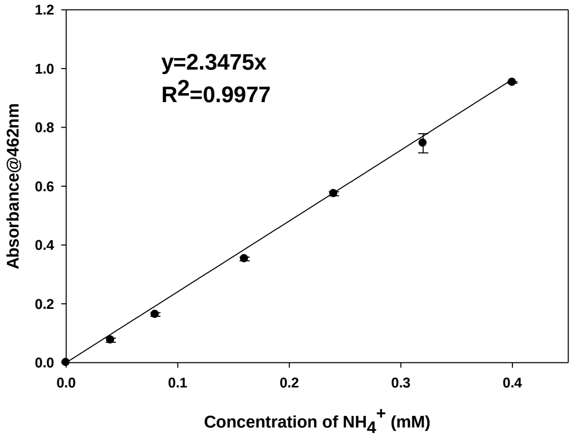

Supplementary Figure 25. The standard curve for NH_4^+ titration. The standard solution was prepared by 2 mM $(NH_4)_2SO_4$. The EgtE reaction sample provided the reading of 0.7881 ± 0.0035. At the same time, a standard curve was generated using NH_4^+ solutions and used to calculate the amount of NH_4^+ produced from EgtE reaction. Based on our measurement, the ratio between formed NH_4^+ and the ergothioneine is ~ 1:1.

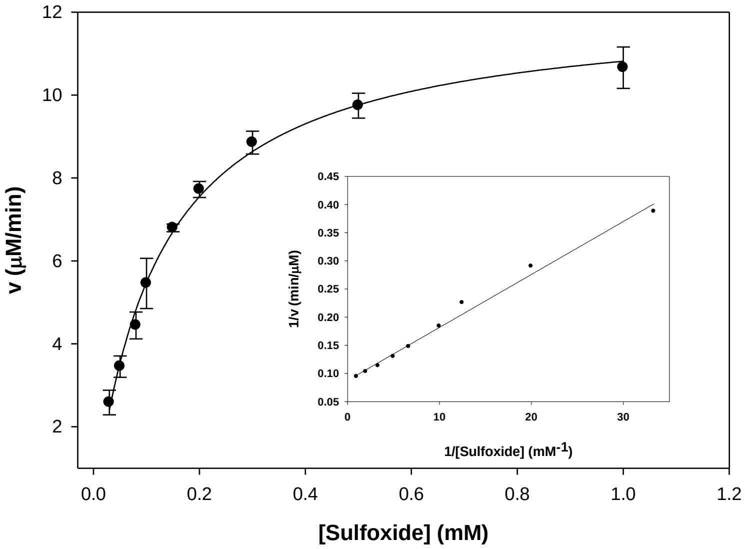

Supplementary Figure 26-1. EgtE steady-state kinetic analysis at 25 °C. Assay mixtures contained 10 nM of EgtE, 0.13 mM NADH, 1 mM DTT, 22.5 U/mL LDH (2000× relative to EgtE activity in the assay) in 50 mM KPi buffer, pH 8.0 and various amounts of sulfoxide **4** in a total volume of 1.0 mL with stirring. The reaction was monitored at 340 nm using the Varian Cary 100 Bio UV-vis spectrometer. The data was fitted by SigmaPlot.

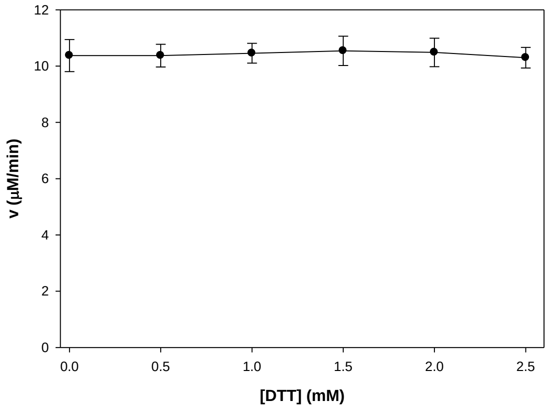

Supplementary Figure 26-2. DTT effect on EgtE activity at 25 °C. Assay mixtures contained 10 nM of EgtE, 0.13 mM NADH, 1 mM sulfoxide **4**, 22.5 U/mL LDH (2000× compared to EgtE activity in the assay) in 50 mM KPi buffer, pH 8.0 and various amounts of DTT in a total volume of 1.0 mL. The reaction was monitored at 340 nm using the Varian Cary 100 Bio UV-vis spectrometer. The data was fitted by SigmaPlot.

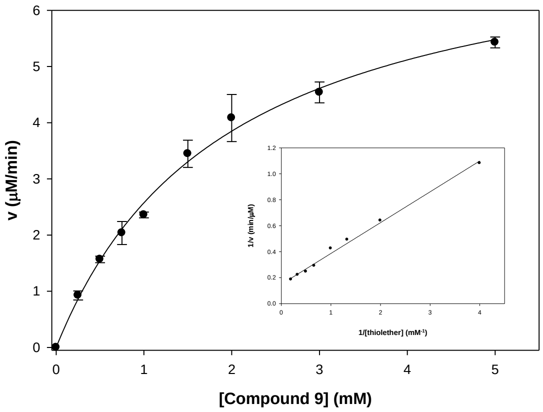

Supplementary Figure 26-3. EgtE steady-state kinetic analysis with using thio-ether **8** as substrate at 25 °C. Assay mixtures contained 20 nM of EgtE, 0.13 mM NADH, 1 mM DTT, 22.5 U/mL LDH (at least 1000× compared to EgtE activity in the assay) in 50 mM KPi buffer, pH 8.0 and various amounts of thio-ether substrate **8** in a total volume of 1.0 mL with stirring. The reaction was monitored at 340 nm using the Varian Cary 100 Bio UV-vis spectrometer. The data was fitted by SigmaPlot.

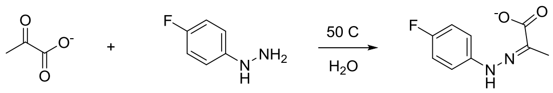

pyruvate (**21**) 4-Fluorophenylhydrazine (**22**) **23**

Supplementary Figure 27. Derivatize pyruvate using 4-fluorophenylhydrazine (**22**).

Supplementary Methods

Expression and purification of EgtE. The EgtE gene (GenBank: ABK70212.1) from *Mycobacterium smegmatis* str. MC2 155 was sub-cloned into the EcoRI and XhoI sites of pASK-IBA3+ expression vector from IBA GmbH.

The sequence of the recombinant EgtE is:

MGDRGPEFLAQQWRDARPKVAGLHLDSGACSRQSFAVIDATTAHARHEAEVGGYVAAEAATPALDA
GRAAVASLIGFAASDVVYTSGSNHAIDLLLSSWPGKRTLACLPGEYGPNLSAMAANGFQVRALPVDDD
GRVLVDEASHELSAHPVALVHLTALASHRGIAQPAAELVEACHNAGIPVVIDAAQALGHLDCNVGADA
VYSSSRKWLAGPRGVGVLAVRPELAERLQPRIPPSDWPIPMSVLEKLELGEHNAAARVGFSVAVGEHL
AAGPTAVRERLAEVGRLSRQVLAEVDGWRVVEPVDQPTAITTLESTDGADPASVRSWLIAERGIVTTA
CELARAPFEMRTPVLRISPHVDVTVDELEQFAAALREAPLEVDLQGDHGLSAWSHPQFEK

The amino acids colored in red are extra amino acids introduced during the sub-cloning process. The amino acid sequence colored in blue is the Strep-tag, which is used for affinity-based purification by Strep-Tactin resin.

The EgtE-pASK-IBA3$^+$ construct was transformed into BL21(DE3) cell and grown in 4L LB medium (supplemented with 0.1 mM PLP and 100 μg/mL ampicillin). After the OD_{600} reached 0.6, anhydrotetracycline (AHT) was added to a final concentration of 200 μg/L to induce EgtE overexpression at 16 °C for 16 hours. Cells (10g) were resuspended in 50 mL of buffer (100 mM Tris-HCl, 50 mM NaCl, pH 7.5). Lysozyme (1.0 mg/mL of final concentration) and DNase I (100 U/g cell) were then added into the cell suspension and the mixture was incubated on ice for 40 min with gentle agitation. The cells were disrupted by sonication (20 cycles of 30 s bursts). The supernatant and the cell debris were separated by centrifugation at 4 ^{o}C for 10 min at 20,000 g. To the supernatant (50 mL), streptomycin sulfate was added to a final concentration of 1% (w/v %) and the mixture was incubated on ice for 30 min with gentle agitation. The white DNA precipitate was then separated by centrifugation at 20,000 g for 30 min at 4 °C. The resulting supernatant was mixed with the Strep-Tactin resin (10 mL) and incubated on ice for 30 min. After the cell lysate was drained by gravity, the column was washed with washing buffer (100 mM Tris-HCl, 50 mM NaCl, pH 7.5) until the OD_{260} is lower than 0.05. The recombinant protein was eluted with the elution buffer (2.5 mM desthiobiotin in 100 mM Tris-HCl buffer, pH 7.5). After the protein was concentrated by ultrafiltration, it was flash frozen by liquid nitrogen and stored at -80 °C. The typical yield is ~ 2 mg of purified EgtE per gram of wet cells.
